# The role of cancer stromal fibroblasts in mediating the effects of tobacco-induced cancer cell growth

**DOI:** 10.1186/s12935-021-02414-9

**Published:** 2021-12-25

**Authors:** Zai-Zai Cao, Yin-Jie Ao, Shui-Hong Zhou

**Affiliations:** Department of Otolaryngology, The First Affiliated Hospital, College of Medicine, Zhejiang University, No. 79, Qinchun Road, Shangcheng District, Hangzhou, 310003 Zhejiang China

**Keywords:** Tobacco products, Cancer, Fibroblasts, Carcinogenesis

## Abstract

Tobacco products cause a variety of cancers, nicotine and carcinogens are two major factors to link the tobacco products and various cancers. The mechanism of tobacco inducing carcinogenesis and promoting cancer progression have been studied for a long time. However, mainstream studies just focus on the mutagenic characteristics of tobacco product and its properties to induce carcinogenesis of epithelial cells. In the past decades, people began to aware of the significant role of tumor stroma in cancer development and progression. Fibroblasts, which is associated with various cancer in all stage of disease progression, are the dominant cell type in the tumor microenvironment. While only a few studies explore the crosstalk between tobacco-induced fibroblasts and surrounding epithelial cells. Our purpose is to systematically review the effects of tobacco products on fibroblasts and further discuss how these effects affect the development of cancer cells.

## Background

At present, consumers all over the world are using many different tobacco products, such as factory produced tobacco, pipe tobacco, snuff and e-cigarette. Using tobacco product have a history of hundreds of years in the world, and evaluation of epidemiologic data has shown that about one-third of people around the world are using tobacco products [1, 2]. Smoking does great harm to the human body, exposure of tobacco smoke has been thought as a significant risk factor for multiple diseases, such as cardiovascular disease, chronic obstructive pulmonary disease and cancer [1]. Scientists and tobacco manufacturers have been focusing on reducing the harm of tobacco for many years. The emergence of electronic cigarette seems to bring a glimmer of dawn to human health, however, the chemicals contained in E-cigarettes have not been fully understand [3]. Nicotine and various levels of toxic chemicals (acetaldehyde, formaldehyde, acetone, acrolein, chromium, N-nitrosamines, and others) are potential health risks of E-cigarettes [4]. Previous studies have shown that the occurrence of at least 12 kinds of cancers were related to tobacco smoking (lung, head and neck, liver, pancreas, esophagus, bladder, kidney, etc.) [4, 5] and there are more than 70 carcinogens in cigarette smoke while at least 16 carcinogens in unburned tobacco [6]. Among all these carcinogens, tobacco-specific nitrosamines such as NNK (4-(methylnitrosamino)-1-(3-pyridyl)-1-butanone) and NNN (N’-nitrosonornicotine), polycyclic aromatic hydrocarbons and aromatic amines are considered to play the most important role in malignant transformation. DNA damage and adduct formation are thought to be the major mechanism by which these compounds cause mutations and drive the carcinogenic transformation of the epithelial cells [7].

As everyone knows, tumor stroma plays an important role in cancer development and progression [8]. Tumor stroma, which is mainly composed of extracellular matrix (ECM), fibroblasts, endothelial cells and immune cells, plays a significant role in leading the complex cell–cell or cell-ECM interactions that contribute to cancer development. Fibroblasts are the main cell type in stroma and are considered to play a key role in both normal homeostasis and wound healing. In steady state, fibroblasts can modulate the homeostasis of epithelial cells through both direct and in-direct interaction [9]. While fibroblasts were activated, normal tissue fibroblasts were able to acquire myofibroblast state (expression of α-SMA) and play an important role in angiogenesis and epithelial proliferation. Microenvironment of cancer was ever described as “wounds which do not heal” [8], Thus cancer associated fibroblasts (CAFs) are similar to myofibroblasts in many ways. During the development of cancers, CAFs can promote the proliferation and metastasis by changing the components of the ECM, forming paracrine signaling loops with cancer cells, inducing metabolic reprogram in surrounded epithelial cells and even regulating the tumor immunology [10]. Recent studies have shown that tobacco exposed fibroblasts can also obtain myofibroblast phenotype and affect the proliferation and migration of epithelial cells. Therefore, one of our aims was to review the effects of tobacco products on fibroblasts and clarify the difference among normal fibroblasts, CAFs and tobacco-exposed fibroblasts [11]. The effect of tobacco on different tumor has been extensively reported previously. However, most research just focus on the mutagenic properties of tobacco products and its ability to induce mutations that necessary for tumorigenesis. Only a few studies focus on the association of fibroblasts in the tumor stroma with smoking mediated carcinoma progression. Therefore, On the basis of clarifying the effects of tobacco products on fibroblasts, we would like to further review how these effects influence the development of surrounding epithelial cells or cancer cells.

## The effect of tobacco product on fibroblasts

**Fig. 1:**
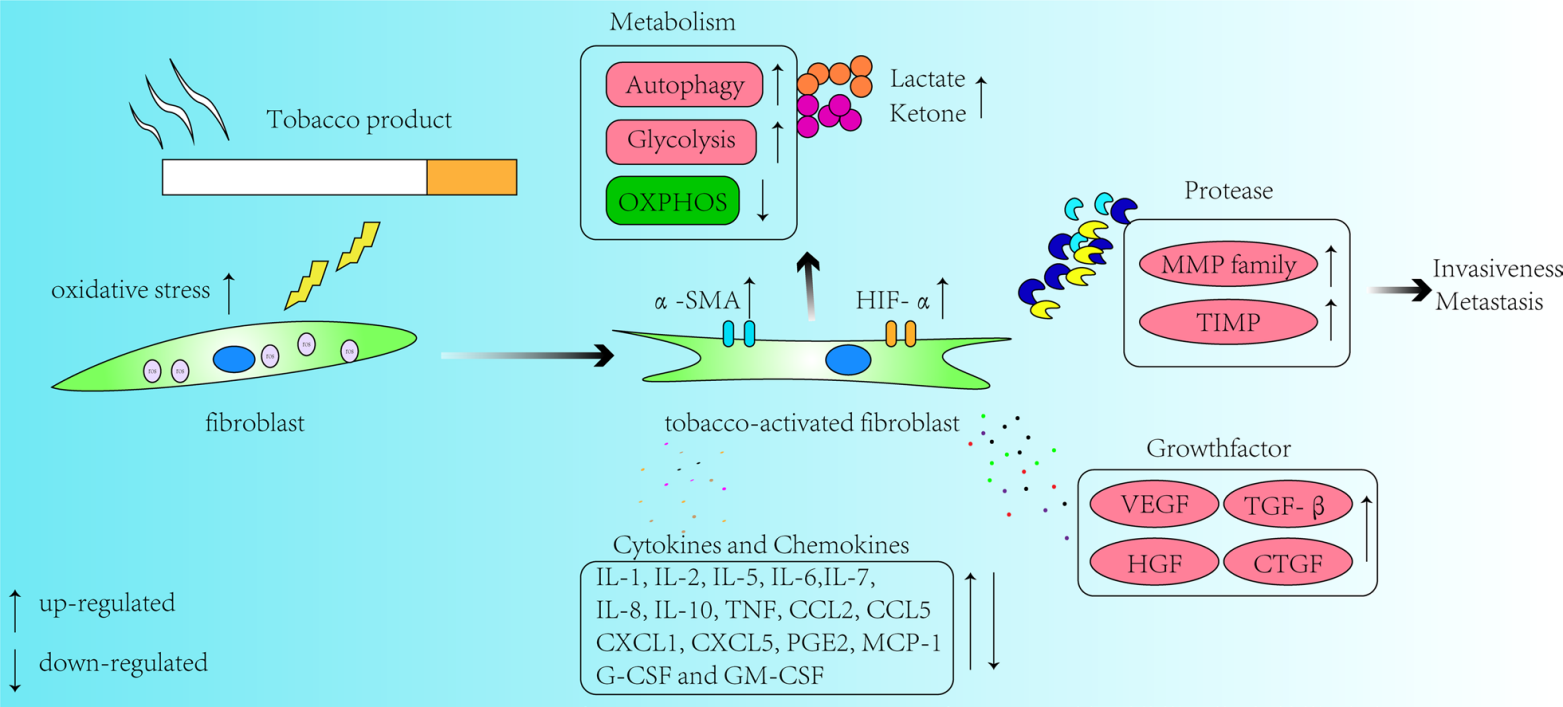
Effects of tobacco product exposure on fibroblasts. Tobacco products can activate fibroblasts to a certain extent and promote myofibroblastic differentiation of fibroblasts, thus leading to a series of changes in cells function (Glucose metabolism, Oxidative stress, Protease secretion, Growth factor secretion and Cytokine secretion)

Previous studies have shown that tobacco products have multiple effects on the phenotype, development and function of fibroblasts. We reviewed a series of previous studies to explore the possible effects of tobacco products on fibroblasts (Fig. 1).

### Tobacco product and oxidative stress in fibroblast

Oxidative stress is one of the important causes of abnormal cell function caused by tobacco products. A great number of reactive oxygen species (ROS) were presented in fibroblasts under the stimulation of tobacco products thus leading to Imbalance between oxidation and antioxidation in vivo. oxidative stress always involved in various biological and pathological processes, like inflammation and tumorigenesis. A great number of studies have confirmed that tobacco products can induce oxidative stress and promote apoptosis in human fibroblasts [12–15]. High concentrations of oxidants, which leads to excessive oxidative stress product (H2O2, NO, CO, Lipid peroxidation products, et al.) formation in cells, included in tobacco product Is the main cause of oxidative stress [15, 16]. Besides, Cigarette smoke extracts elevated HIF-1α levels [17], which can further lead to an induction of oxidative stress and create a pseudo-hypoxic state [18]. On the other side, oxidative stress caused by tobacco products also significantly affect the activity of anti-oxidative enzymes and impair the defense system of ROS. Lee et al. showed that CSE (Cigarette smoke extract) treatment markedly decreased the level of glutathione and weakened the activity of SOD (superoxide dismutase) in lung fibroblasts [19], thus promoting the progression of oxidative stress. At relatively lower concentrations, ROS are important signaling molecules involved in cellular proliferation, migration, and apoptosis [20, 21]. However, the accumulation of ROS can cause Irreversible damages to the double-stranded DNA and changes to proteins and lipids, thus finally leading to a serious of detrimental effects, such as death, mutagenesis, tumorigenesis and fibrosis [21].

### The effect of tobacco product on the contractility, proliferation and migration of fibroblasts

The production of a large amount of ROS is one of the most important reasons that tobacco products affect the phenotype of fibroblasts. Previous reports showed that oxidative stress caused by tobacco product in human lung, gingival or nasal fibroblasts are thought to contribute a lot in attenuates collagen gel contraction and wound healing [15, 22, 23]. Shin et al. showed that ROS/AMPK is the downstream signal pathway respond to oxidative stress caused by tobacco products [22]. The use of antioxidants can remarkably promote proliferation and migration on human fibroblasts [24]. Although major studies showed that tobacco product is a potent inhibitor of fibroblast functions including cell proliferation, migration and contractility [23], Silva et al. point out that the concentration of tobacco exposure will affect the effect of tobacco on fibroblasts. At low concentrations, CSC (cigarette smoke condensate) can increase cell survival, promoted migration. However, the above cell responses would be inhibited when exposure to a higher level of CSC [25]. Yang et.al showed that proliferative capacity and growth of fibroblasts were inhibited by CSE exposure in a dose- and time-dependent manner [24]. However, the mechanism of the above phenomenon remains unclear. Besides, CSE also promotes the apoptosis of lung fibroblasts through phosphorylate STAT1 and up-regulate the MAPK pathway, thus inhibiting the proliferation and migration property [19].

### Tobacco product and myofibroblasts differentiation of fibroblast

Myofibroblasts are a group of heterogeneous cells that responsible for ECM secretion and tissue contraction. The expression of α-SMA is the main marker of matured myofibroblasts. The role of tobacco products in myofibroblasts differentiation is controversial. Experimental design, origin of fibroblasts, Reagent difference and type of tobacco product may contribute to this controversial. A variety of toxic substances that originated from tobacco can promote myofibroblastic differentiation in multiple ways. Virender et al. suggested that nicotine induces Human embryonic lung fibroblasts-to-myofibroblasts transdifferentiation through a mechanism involving downregulation of lipogenic human parathyroid hormone-related protein (PTHrP)-mediated, cAMP-dependent PKA signaling pathway [26]. In addition to nicotine, other carcinogenic components of tobacco (NNK and NNN) have similar ability to promote myofibroblastic differentiation compare with nicotine [27]. Cigarette smoke can also activate surrounding epithelial cells and alter the secretion of TGF-β1 or release of extracellular vesicles to promote myofibroblasts differentiation [28, 29]. Moreover, oxidative stress induced by tobacco also contributes to myofibroblasts differentiation. A recent study showed that CSE promotes lung fibroblast-to-myofibroblast differentiation through inducing stress in endoplasmic reticulum [30]. Some other studies showed that nicotine and cigarette smoke inhibits the myofibroblast differentiation induced by TGF-β1 [25, 31, 32], possibly because nicotine disrupts OXPHOS (Oxidative Phosphorylation) in human fibroblasts [31].

### Cigarette smoke could promote the autophagy in fibroblasts

Autophagy, the process by which cells degrade specific auto-cytoplasmic protein or organelles, is one of the characteristics of myofibroblastic differentiation [33]. Some scholars point out that autophagy is a significant mechanism in the interactions between fibroblasts and tumor cells. A previous study has shown that the development of cancer cells may highly depend on the autophagy process in tumor stromal [34]. Multiple studies have explored the mechanism of tumor stromal autophagy promoting cancer cell growth. For instance, autophagy in CAFs can promote the process of energy transportation thus fueling the growth of tumor [35]. Besides, cancer cells can promote autophagy in fibroblasts and induce the release of inflammatory cytokines such as IL6 and IL8 which in turn promote the growth of tumor [36]. Among previous studies, we have observed tobacco induced autophagy in different kinds of fibroblasts like CAFs of breast cancer [37] and human lung fibroblasts [38]. Hou et al. verified that exposed to cigarette smoke extract can up-regulate the expression of autophagy-related protein (LB3 and p62) in fibroblasts and promote the secretion of IL8 [38]. It seems that autophagy in stromal fibroblasts caused by external stimuli could also affect the development of cancer cells.

### Metabolism reprogram induced by tobacco products

Metabolic reprogramming of glycolysis metabolism is also a key event of myofibroblast transition. Recent studies showed that cancer associated fibroblasts (CAFs) have a reprogrammed metabolism with high glycolytic flux, autophagy and senescence, which are cellular processes that provide a nutrient-rich environment for cancer cells [39]. The reprogrammed metabolism induced by tobacco products in fibroblast is similar to which of CAFs. Cigarette smoke extract exposure increases glycolysis rates, downregulates the expression of OXPHOS complexes, promotes the secretion of L-lactate and ketone bodies as well as inducing senescence in fibroblasts [7, 37]. Tobacco products trigger oxidative stress, which leads to mitochondrial dysfunction in fibroblasts, contributes a lot to reprogram progression. Salem et al. pointed out that genetic and environmental factors can promote the induction of CAFs phenotype through common mechanisms [37], For instance, breast carcinoma cell lines also induce the CAFs phenotype through modulating oxidative stress and glucose metabolism in the fibroblasts which is same as ethanol or tobacco exposure [40, 41].

### Tobacco products affect the secretion of proteases in fibroblast

Compared with normal fibroblasts, the secretory phenotype of tobacco-activated fibroblasts also undergoes a series of changes. MMPs are significant members of ECM-degrading proteases, which are mainly originated from myofibroblasts. Numerous studies showed that tobacco can promote the expression of MMPs (matrix metalloproteinase) (MMP1, MMP2, MMP3, MMP 8, MMP9, MMP14) [42–45] and TIMP (Tissue inhibitor of metalloproteinases) in fibroblasts [45, 46]. Migration and invasion of tumor cells are facilitated by MMPs. Among the MMPs family, MMP3, which also known as Stromelysin 1, is secreted robustly by activated fibroblasts and cleaves E-cadherin, thus prompting the process of EMT and invasiveness in surrounding tumor cells [46]. MMP1 and MMP2 were also proved to induces invasiveness of surrounding epithelial cells [47, 48]. The mechanism of tobacco-induced MMPs expression in fibroblasts is not clear. studies revealed that the AhR, HIF-1α and ERK1/2 may be the target of nicotine to promote the overexpression of MMPs [49–51].

### Tobacco products affect the secretion of growth factor in fibroblast

Growth factors are a class of peptides that regulate multiple effects such as cell growth and other cell functions by binding to specific cell membrane receptors. Although there is direct experimental evidence showed that tobacco product can alter the secretion of various growth factor, including (TGF)-β, CTGF (Connective tissue growth factor), VEGF (Vascular endothelial growth factor) and HGF (Hepatocyte growth factor) [13, 32, 52, 53], the mechanism is not very clear. The study demonstrated that fibroblasts have exaggerated response to cigarette smoke extract along with increased oxidative stress and growth factor (CTGF and TGF-β), the use of antioxidants can significantly reverse the increased growth factor induced by CSE [13], indicating that Oxidative stress plays an important role in this progression. Shin et al. also showed that ROS can mediate cigarette smoking induced VEGF secretion in nasal fibroblasts [54]. MAPK/NF-κB pathway was reported to mediate the secretion of growth factor in CSE altered fibroblast [53–55]. Ginsenoside Rb3 exerts can help prevent fibroblasts injury from cigarette smoke and alter the secretion of TGF-β1 and VEGF through inhibiting the MAPK/NF-κB pathway [53]. In addition, cigarette smoke alters the expression of Cyr61, a member of the CTGA, via Egr-1 in human skin dermal fibroblasts [56]. The altered TGF-β, CTGF, VEGF and HGF acts by altering proliferation, migration, invasiveness, angiogenesis, and drug resistance leading to carcinogenesis and cancer progression. TGF-β also reported to regulates a myriad of mainly immunosuppressive responses in tumor, however, no research linking the tobacco altered TGF-β to immune response of cancer cells directly. We need more research to help us clarify the role of tobacco altered growth factor across a multitude of human cancers.

### Tobacco products affect the secretion of cytokines and chemokines in fibroblasts

The secretion of cytokines and chemokines can alter tumor immunity at various stages of cancer development. Although tobacco products can alter the secretion of various chemokines or cytokines in fibroblasts, tobacco induced secretion phenotypes are heterogeneous in different fibroblasts. It is therefore challenging to define how tobacco products alter the secretome of fibroblasts in vivo. Almost no research in the past directly linking tobacco altered secretomes to immune responses of surrounding epithelial cells. Directly evidence showed that tobacco product can alter the secretion of numerous cytokines and chemokines including, but not limited to, IL-1, IL-2, IL-5, IL-6,IL-7, IL-8, IL-10, TNF, CCL2, CCL5, CXCL1, CXCL5, PGE2, MCP-1, G-CSF and GM-CSF [57–60]. However, the role of tobacco in changing secretory phenotype is different among different fibroblasts. For instance, two studies showed that cigarette smoke condensate can decrease the secretion level of IL-6, IL-8 and TNF in dermal or gingival fibroblasts [61, 62]. However, In lung fibroblasts, studies showed that cigarette smoke can promotes the secretion of the above cytokines through MAPK pathway [19, 63]. Among all cytokines and chemokines that altered by tobacco products, some have been reported to play a significant role in tumor immunity. For example, IL-6 signaling has been showed to restrict the maturation of DCs, inhibit the activation of T cells and inducing T cells anergy [64], fibroblast-originated IL-6 also redirects monocytes towards differentiation into a macrophage lineage rather than DC differentiation [65]. Fibroblasts-derived IL-4, IL-6 and IL-8 may induce immunosuppressive myeloid cell differentiation [66]. CCL2 can recruit inflammatory monocytes to promote the metastasis of breast cancer [67]. Although no direct evidence showed that tobacco products altered secretomes can affect the tumor immunity, ongoing and future studies may fill this blank.

## Exposure to tobacco can alter the interaction between fibroblast and surrounding epithelial cells

**Fig. 2:**
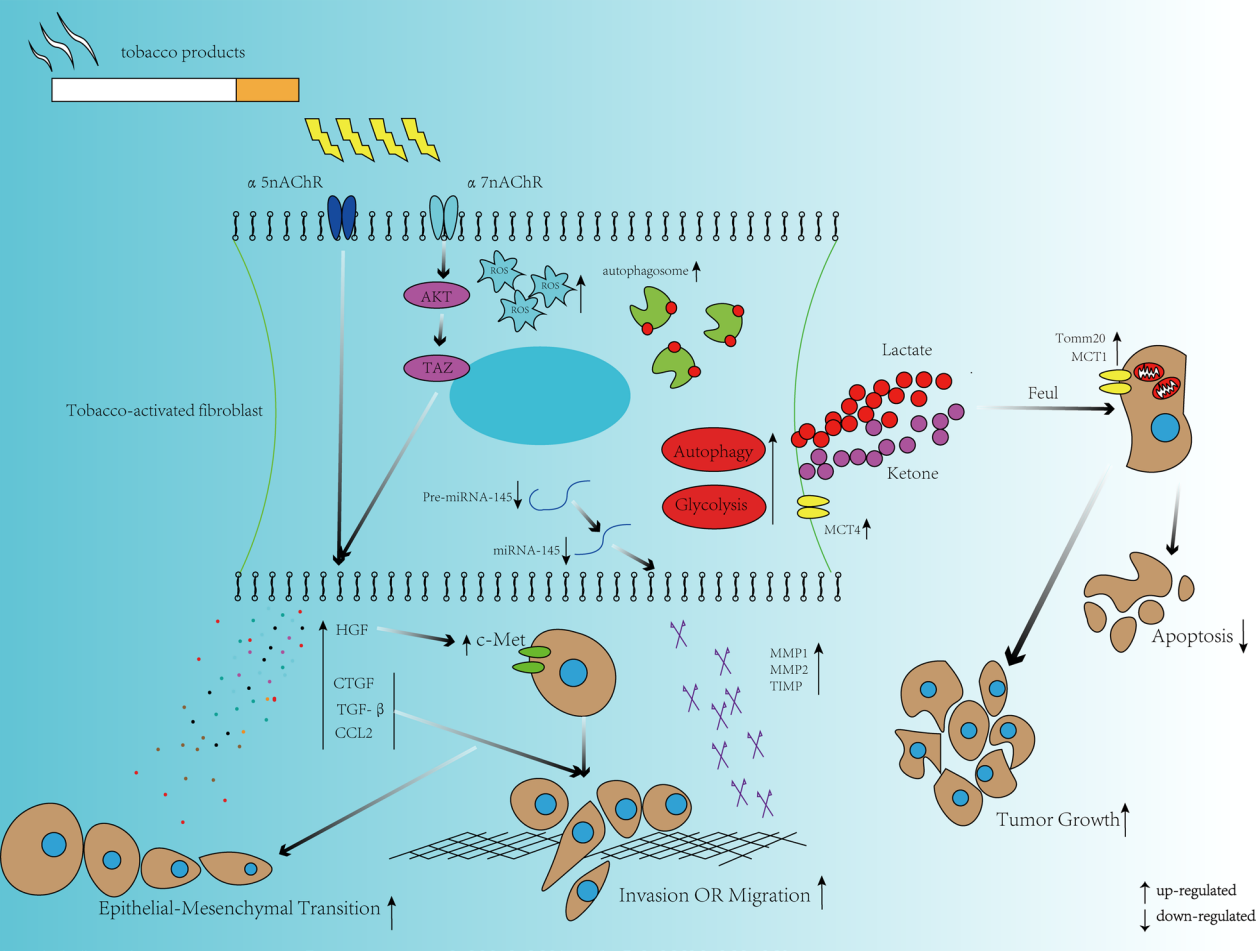
The potential mechanism of tobacco-activated fibroblasts leading to carcinogenesis and cancer progression. Tobacco products can directly or indirectly stimulate fibroblasts to make a series of changes in their internal functions and signaling pathways. These changes will have a series of effects on the phenotype of peripheral epithelial cells (brown). For instance, growth factors released by tobacco-activated fibroblasts can promote the migration and invasion of peripheral cancer cells and induce epithelial-mesenchymal transformation. Tumor cells can use the high-energy metabolites produced by tobacco-activated fibroblasts for their own growth. Besides, one report showed that tobacco can promote metastasis of oral squamous cancer cells by suppressing miR-145 in fibroblasts

While the effect of tobacco on isolated fibroblasts has been studied for a long time, some studies began to focus on how these altered fibroblasts affect the microenvironment and epithelial cells in proximity. Almost all current research indicated that tobacco-activated fibroblasts can better promote the carcinogenesis or cancer progression compare with non-tobacco-activated fibroblasts (Table 1). Coppe et al. first reported that Soluble Factors Secreted by Tobacco-Exposed Fibroblasts promote proliferation and interstitial invasion in nonmalignant keratinocyte cell lines but not in normal human primary oral or skin keratinocytes, which indicated that preneoplastic changes in epithelial cells are necessary to sensitize them to stimulation by tobacco-altered fibroblasts. Besides, STE-altered-fibroblasts can decreased the expression of cell polarization and keratinization markers (ZO-1, E-cadherin,β-catenin and Involucrin) in surrounding immortalized epithelial cells [68, 69]. The above phenomenon has been validated in other vitro or vivo models, however, its internal mechanism was only explored in a few studies (Fig. 2).

**Table1:** Studies focus on how tobacco altered fibroblasts affect the microenvironment and epithelial cells in proximity

Author	Year	Type of epithelia cells	Type of fibroblast	Treatment	Main result
In Vitro experiment					
Marina [7]	2019	human head and neck carcinoma cells (CAL27,FaDu)	Mouse Embryonic Fibroblasts Normal human fibroblasts from skin	CSE	1. CSE induces senescence and glycolysis in fibroblasts and CSE exposed fibroblasts can promote mitochondrion OXPHOS in head and neck carcinoma cells 2. Co-culture with CSE-fibroblasts increases features of tumor aggressiveness and proliferation 3. MCT4 expression in tumor stroma is associated with the prognosis of head and neck cancer
Chen [11]	2017	human and breast cancer cell lines (MCF-7, MDA-MB-231)	human embryonic lung fibroblast cells (WI38)	nicotine	1. Nicotine induces myofibroblastic differentiation and Nicotine-treated fibroblasts promote the EMT of breast cancer cells 2. Secretion of CTGF and TGF-β from nicotine-treated fibroblasts enhances breast cancer migration 3. Nicotine induces expressions of CTGF and TGF-β through an α7 nAChR-dependent AKT/TAZ signaling mechanism
Daniel [69]	2016	Human paracrine cancer cell lines (PANC-1, Mia-PaCa-2, BxPC3)	tumor associated fibroblast of pancreatic cancer	nicotine	1. Nicotine treatment augments HGF-MET-mediated paracrine signaling between tumor associated fibroblasts and pancreatic cancer cells, thus promoting tumor growth and metastasis 2 The expression of phosphorylated c-Met directly correlates with reduced overall survival in pancreatic cancer
Melling [43]	2013	oral squqmous carcinoma cell line (SCC4, H357)	primary normal oral fibroblasts	CSC	1. CSC induces changes in miRNA expression in oral fibroblasts and. miR-145 re-expression reverses CSC-induced OSCC chemotaxis
Salem [36]	2013	human triple-negative breast cancer (MDA-MB-231)	Human immortalized fbroblasts (hTERT-BJ1)	CSE	1. CSE induces senescence and DNA damage in stromal fbroblasts by activating the p53-p21-pRb pathway 2. CSE treatment promotes autophagy and mitophagy, downregulating the expression of mitochondrial OXPHOS complexes in fibroblasts 3. CSE-treated fbroblasts produce high levels L-lactate and ketone bodies, indicative of mitochondrial dysfunction: A shift toward glycolysis and ketogenesis
Coppe [68]	2008	two nonmalignant keratinocyte cell lines (DOK,HaCAT) normal human epidermal keratinocytes (NHEK; Cambrex) oral squamous cell carcinoma cell lines (HSC-3)	Normal human fibroblasts from skin Normal human fibroblasts from oral mucosa Normal human fibroblasts from embryonic lung	STE	1. STE promote proliferation of fibroblasts and Induce ROS production and oxidative DNA damage in fibroblasts 2. STE alter the secretory phenotype of fibroblasts thus stimulating proliferation of skin and oral keratinocytes 3. STE–exposed fibroblasts stimulate Interstitial Invasion of oral epithelial cells and down-regulate cell polarization and keratinization markers
Hou [37]	2020	Nonsmall lung cancer cell line (CL1-0)	human lung fibroblast cell line (MRC-5)	CSE	1. CSE-treatment promotes autophagy in fibroblasts 2. CSE-treatment fibroblasts promote the invasion of cancer cells in 2D and 3D model with secretion of IL-8
In Vivo experiment					
Marina [7]	2019	human head and neck carcinoma cells (CAL27,FaDu)	Athymic nude mice	CSE	1. Co-injection of carcinoma cells with CSE-fibroblasts increases tumor growth
Salem [36]	2013	human triple-negative breast cancer (MDA-MB-231)	Athymic nude mice	CSE	1. CSE-treated fbroblasts enhance tumor growth, independently of neo-angiogenesis
Daniel [69]	2016	surgically resected primary pancreatic adenocarcinoma	Athymic nude mice	nicotine	1. Physiologic doses of nicotine significantly promote the growth and metastasis of tumor 2. Physiologic doses of nicotine induced activation of c-Met within the tumor microenvironment

The change in the production of cytokine secreted by tobacco treated fibroblasts could be a significant factor in promoting carcinogenesis and cancer progression. Daniel et al. showed that nicotine induces HGF-MET signaling among the tumor stroma to promote the pancreatic cancer progression, metastasis and gemcitabine chemoresistance [70]. Similar phenomenon was found in lung cancers. Francesca et al. report that primary fibroblast cell lines derived from lungs of heavy smokers express reduced miR-16 levels compared to those from normal lungs, and reduced expression of miR-16 can upregulate the secretion of HGF, thus affecting the pro-tumorigenic potential of fibroblasts [71]. In addition, altered secretion of CTGF and TGF-β derive from nicotine-exposed-fibroblasts also significantly enhances the migration and EMT of breast cancer and Nicotine may modulate the expressions of CTGF and TGF-β by activating α7 nAChR-dependent AKT/TAZ signaling [11].

Except for the change in cytokine secretion, tobacco exposure can also induce metabolic reprogramming in fibroblast, directly fueling mitochondrial metabolism (OXPHOS) in surrounding tumor cells, actively promoting anabolic tumor growth. The study showed that CSE exposure stimulates autophagy and mitophagy, decreases the expression of mitochondrial OXPHOS complexes in fibroblasts which leading to high secretion level of L-lactate and ketone bodies, indicating a shift toward glycolysis and ketogenesis [7, 37]. What’s more, the conditioned media from CSE treated fibroblasts were able to induce MCT1 and TOMM20 (markers of mitochondrial metabolism) expression in breast cancer cell lines [7], indicating that CSE induced fibroblasts can further reprogram metabolism in surrounding epithelial cells.

Only a small group of mi-RNA are capable of inducing significant phenotypic changes in tumor cells, such as those leading to EMT, which is considered as a key step in cancer invasive and metastasis [72, 73]. Melling etal. for the first time used Tiling low-density array (TLDA) to explore the role of miRNA in modulating the phenotype of fibroblasts in response to tobacco smoke and revealed dramatical changes in mature miRNA expression between CSC-treated-fibroblasts and controlled fibroblasts. Further exploration showed that CSC can promote metastasis of oral squamous cancer cells through stromal–epithelial interactions by suppressing miR-145 [48].

## Inhibition of the interaction between tobacco-induced fibroblasts and cancer cells

From previous studies, we have realized the important role of tobacco-exposed fibroblasts in cancer development. In fact, many scholars have emphasized the importance of fibroblasts in the occurrence and development of cancer in the past decade. However, the most interesting aspects of studies of the role of fibroblasts in development of tumor is the potential implementation of therapeutic strategies that directly target cancer cells. For instance, the metabolism cross talk between fibroblasts and cancer cells has been considered as a potential therapeutic target. The researchers found that we can inhibit the proliferation of cancer cells by targeting multiple metabolic processes (Glycolysis, Glutaminolysis, Ketone Bodies and Fatty acid metabolism) in fibroblasts [74]. “Lactate shuttle” mediated by MCT1 and MCT4 is an important part in the metabolic interaction between fibroblasts and cancer cells. Marina et al. showed that when MCT4 was knocked out, the tumor promoting effect of tobacco exposed fibroblasts seemed to be weakened [7]. Interfering with the role of growth factors in the tumor microenvironment is also considered to be an important therapeutic strategy. A certain degree of tobacco exposure promotes the secretion of a variety of growth factors in fibroblasts like HGF, VEGF and TGF-β. Actually, Anti-TGF-β therapies have been used in clinical trials for various solid tumor [75]. Unfortunately, This treatment seems to induce both antitumor and tumor promoting effects [76, 77]. In another study, Daniel et al. pointed out that when HGF-MET signaling pathway was inhibited, the tumor promoting effect of nicotine exposed fibroblasts was also weakened [70].

## Conclusion and future consideration

Our review indicated that the function and phenotype of fibroblasts can be altered by various tobacco products, thereby changing the metabolism of fibroblasts, the secretion of growth factors and the construction of extracellular matrix to self-regulate their expansion, regulate inflammation, immunity. Under the milieu of such circumstances, the adjacent epithelial cells can therefore acquire advantageous growth, migratory, survival even cancerous properties from the released metabolite and growth-promoting factors. In the past, when exploring the carcinogenic mechanism of tobacco products, researchers often focused on the direct effect of tobacco products on tumor cells. Our review highlights the important role of fibroblasts in tobacco induced carcinogenesis. Actually, in addition to fibroblasts, tobacco products can also affect a variety of immune cells. Previous studies have shown that tobacco products impacts both innate (DCs, macrophages and NK cells.) and adaptive (T helper cells, regulatory T cells, CD8 + T cells and B cells) immunity cells and play crucial role in regulating immunity by either strengthen pathogenic immune response or weaken defensive immunity. However, to our knowledge, although tobacco products have a series of effects on immune cells, few studies have explored how these effects affect the development of tumor cells. This research gap should be filled in the future. All in all, when discussing the carcinogenic mechanism of tobacco products, we should pay more attention to the role of tumor microenvironment.

## Data Availability

Not applicable.

## Abbreviations

ECM: Extracellular matrix
NNK 4: (Methylnitrosamino)-1-(3-pyridyl)-1-butanone
NNN: N’-nitrosonornicotine
SOD: Superoxide dismutase
ROS: Reactive oxygen species
CSE: Cigarette smoke extract
MMP: Matrix metalloproteinase
TIMP: Tissue inhibitor of metalloproteinases CSC cigarette smoke condensate
STE: Smokeless tobacco extracts
TGF: Transforming growth factor
CTGF: Connective tissue growth factor
VEGF: Vascular endothelial growth factor
HGF: Hepatocyte growth factor
TNF: Tumour necrosis factor
CCL: C–C motif chemokine ligand
CXCL: C-X-C motif chemokine ligand
PGE2: Prostaglandin E2
MCP: Monocyte chemoattractant protein
G-CSF: Granulocyte colony-stimulating factor
GM-CSF: Granulocyte–macrophage
IL: Interleukin
DCs: Dendritic cells
EMT: Epithelial-mesenchymal transition
OXPHOS: Oxidative phosphorylation
